# Exploring Fungal Abundance and WHO Fungal Priority Pathogens in Agricultural Fields: A One Health Perspective in Northeast Thailand

**DOI:** 10.3390/life15030488

**Published:** 2025-03-18

**Authors:** Chayaporn Lakmuang, Syahriar Nur Maulana Malik Ibrahim, Teeratat Kaewjon, Nattapol Kraisitudomsook, Naraporn Somboonna, Ratmanee Chanabun, Ariya Chindamporn, Nuttapon Pombubpa

**Affiliations:** 1 Bioinformatics and Computational Biology Program, Graduate School, Chulalongkorn University, Bangkok 10330, Thailand; chayaporn.la@ku.th; 2 Department of Microbiology, Faculty of Science, Chulalongkorn University, Bangkok 10330, Thailand; syahriarmicro@gmail.com (S.N.M.M.I.); teeratat.kj@gmail.com (T.K.); naraporn.s@chula.ac.th (N.S.); 3 Department of Biology, Faculty of Science and Technology, Muban Chombueng Rajabhat University, Ratchaburi 70150, Thailand; nattapolkra@mcru.ac.th; 4 Microbiome Research Unit for Probiotics in Food and Cosmetics, Chulalongkorn University, Bangkok 10330, Thailand; 5 Program in Animal Science, Faculty of Agricultural Technology, Sakon Nakhon Rajabhat University, Sakon Nakhon 47000, Thailand; ratmanee@snru.ac.th; 6 Biodiversity and Utilization Research Unit, Center of Excellence in Modern Agriculture, Sakon Nakhon Rajabhat University, Sakon Nakhon 47000, Thailand; 7 Department of Microbiology, Faculty of Medicine, Chulalongkorn University, Bangkok 10330, Thailand; drariya@gmail.com

**Keywords:** fungal pathogen, WHO FPPL, agriculture, one health, metabarcoding

## Abstract

Fungal pathogens prevalent in agricultural areas pose a significant risk to human health, with some exhibiting high fatality rates, as reported by the WHO Fungal Pathogen Priority List (WHO FPPL). This study investigates fungal communities in northeast Thailand’s agricultural areas, focusing on potential reservoirs of the WHO FPPL. Samples were collected from rice, cassava, rubber trees, and sugarcane fields across 18 provinces with distinct geological features. Metabarcoding of the ITS1 region and taxonomic analysis were conducted, and potential pathogens were selected according to WHO FPPL criteria. The results showed that overall fungal community richness and diversity were influenced by plant fields but not significantly different by geological features. Soil organic matter and water content affected fungal dynamics only in rubber tree fields. Fungal pathogens from the WHO FPPL were found in all four plant fields, with higher abundance in Chaiyaphum province, especially in sugarcane fields, and the lowest in Nong Bua Lam Phu. *Candida tropicalis*, a high-priority pathogen, was predominantly associated with rock salt features. This study underscores the need for vigilance among farmers and emphasizes the importance of confirming fungal pathogenicity.

## 1. Introduction

Recently, the World Health Organization (WHO) announced a fungal priority pathogen list (WHO FPPL) concerning fungal pathogens categorized into a (1) critical priority group, (2) high priority group, and (3) medium priority group [1]. Some of these fungal pathogens have already been reported in tertiary care hospitals in northeast Thailand, including *Candida albicans*, *Cryptococcus neoformans*, and *Talaromyces marneffei*, with candidiasis as a significant case [2]. Moreover, *Candida tropicalis*, *Candida glabrata*, and *Candida parapsilosis*, which are on the WHO FPPL, have been detected as dominant non-*albicans Candida* in hospitals in Thailand and Asia [3,4]. Some of the fungi in the WHO FPPL have been monitored in clinical settings; at the same time, many are neglected such as *Acremonium* spp., *Fusarium* spp., and *Mucor* spp. [2]. Moreover, the number of fungal infections is often underestimated because reports and data are not always available. Because the geographical origins of these fungal pathogens in Thailand are mostly unknown and very few studies have investigated potential environmental pathogen reservoirs, comprehensive environmental surveys and surveillance are highly valuable. According to the GlobalFungi database [5], there are only a few survey studies on environmental fungi in Thailand that mainly focused on the tropical forest habitats. Some potential plant pathogens were reported, but pathogens in the WHO FPPL were never investigated [6,7]. As a result, WHO FPPL-based environmental surveys are needed to monitor and identify these potential pathogen reservoirs.

In the past, many studies of soil pathogenic fungal communities were based on culture-dependent approaches from environments such as soil to investigate pathogenic fungal communities. However, culture-dependent approaches often underestimate the true biodiversity of fungi [8]. Many factors that lead to deciphering fungal biodiversity such as geography, pH, and temperature may not be accounted for in the laboratory setting [9]. Recently, a culture-independent approach has helped us overcome these limitations. With the advent of technologies, the surveillance and monitoring of fungi in many places under different conditions has become more affordable and more convenient. Nowadays, next-generation sequencing (NGS) is used to investigate microbial composition to understand the type of microbes that are present and their potential to cause diseases in humans, plants, and other animals [10]. Thus, NGS technology can potentially be used to identify potential pathogenic fungi and prevent opportunistic infections [11,12].

The northeastern region of Thailand (14–19° N, 101–106° E) consists of 20 provinces including Amnat Charoen, Buri Ram, Bueng Kan, Chaiyaphum, Kalasin, Khon Kaen, Loei, Maha Sarakham, Mukdahan, Nakhon Phanom, Nakhon Ratchasima, Nong Bua Lamphu, Nong Khai, Roi Et, Sakon Nakhon, Sisaket, Surin, Ubon Ratchathani, Udon Thani, and Yasothon. It covers 168,894 km^2^ or one-third of the country and is located on the Khorat plateau, consisting mostly of undulating land [13,14]. Most of the population engages in agriculture activities, especially rainfed agriculture, including rice, cassava, sugarcane, and rubber [13,14,15]. The distribution of those agriculture fields is divided into eight geological feature zones: zone 1 (Loei Petchabun), zone 2 (Phu Wiang), zone 3 (Khorat plateau), zone 4 (Phu Phan), zone 5 (Udon Sakon Nakon), zone 6 (Bueng Kan), zone 7 (Khorat Ubon), and zone 8 (Buriram) [16]. Pathogens from the WHO FPPL identified in hospital studies from this region were recorded between 2006 and 2011, with *C. neoformans* most commonly infecting the central nervous system (84.9%), *Candida* spp. infecting the gastrointestinal (64.3%) and urinary tracts (66.7%), and *Aspergillus* spp. infecting the respiratory tract (31.6%) [2]. Besides hospital reports, there is very limited knowledge about potential fungal pathogens in the northeastern region, especially from the soil. This area might serve as an environmental reservoir for human fungal pathogens because many agriculturalists in Thailand use traditional methods, such as irrigated farming, that require intense, direct human contact with soil and plants [13]. Humans are directly exposed to fungi daily either through inhalation, ingestion, or skin contact [17].

Previous studies in 2014 on fungal infections in northeast Thailand identified pathogens on the WHO FPPL, including two critical-priority, eleven high-priority, and one medium-priority species, which were obtained from a single medical institute using clinical samples with culture-dependent methods [2]. In 2010, critical-to-medium-priority levels of pathogens on the WHO FPPL, including *Aspergillus fumigatus*, Mucorales, *Fusarium*, *Curvularia*, and *Scedosporium apiospermum*, were isolated from house flies in Ubon Ratchathani [18]. Three critical-priority WHO FPPL pathogens were identified in northeastern Thailand using culture-dependent methods; *C. albicans* and *C. neoformans* were identified in clinical settings [19,20], and *A. fumigatus* was identified in rice fields [21]. In 2015, three species of *Candida*—*C. tropicalis*, *C. parapsilosis*, and *Nakaseomyces glabrata* (basionym as *C. glabrata*)—were cultured and identified from clinical samples, all of which are classified as high-priority species within the WHO FPPL [22]. From 1996 to 2002, studies conducted at Srinagarind Hospital in Khon Kaen examined Histoplasmosis caused by *Histoplasma* spp. and Penicilliosis caused by *Talaromyces marneffei* (basionym as *Penicillium marneffei*) [23]. In 2021, a new case of Histoplasmosis emerged, with suspicions that the patient had traveled through areas where soil was contaminated by bird or bat excretions [24]. *Pneumocystis jirovecii* causative of pneumonia were found in clinical and rodent animal samples in northeast Thailand using molecular methods [2,25,26]. Another medium-priority level, *Cryptococcus gattii*, was found in northeast Thailand [27]. While some pathogens on the WHO FPPL have been reported from clinical settings, the knowledge of potential WHO FPPL reservoirs in the agricultural areas of northeast Thailand remains limited. This lack of data and surveillance procedures hampers efforts to raise awareness and provide warnings to farmers [28], which is crucial from a one health perspective [29]. Investigating WHO FPPL communities in the environment presents considerable challenges, such as the difficulties of isolating fungi in the laboratory and the variability of spatial and temporal factors [30]. To address these challenges and enhance our understanding, we utilized next-generation sequencing (NGS) to examine the distribution of WHO FPPL pathogens in northeast Thailand. This approach aims to document potential WHO FPPL reservoirs, providing crucial insights that could help reduce the incidence of infectious fungal diseases in humans, animals, and plants in the future, thus aligning with the one health concept.

Our concerns regarding WHO FPPL pathogen detection are escalating due to the scarcity of environmental data, which is crucial for understanding the interconnected health of humans, animals, and the environment. This project seeks to address this critical research gap by examining fungal diversity in agricultural regions of northeast Thailand. By documenting and analyzing these findings, we aim to provide guidelines for the future exploration of potential pathogenic fungal reservoirs, emphasizing the one health concept. This approach ensures a comprehensive understanding of how fungal pathogens in the environment can impact agricultural productivity, animal health, and human health, ultimately contributing to the prevention and control of fungal infections in Thailand. We hypothesize that (1) fungal richness and communities will be significantly different according to plant fields and geological features, (2) fungal pathogen richness from the WHO FPPL will also be different by plant fields, and (3) the distribution patterns of pathogens on the WHO FPPL will be different for each WHO FPPL pathogen detected in northeast Thailand.

## 2. Materials and Methods

### 2.1. Sample Collection

**Figure 1:**
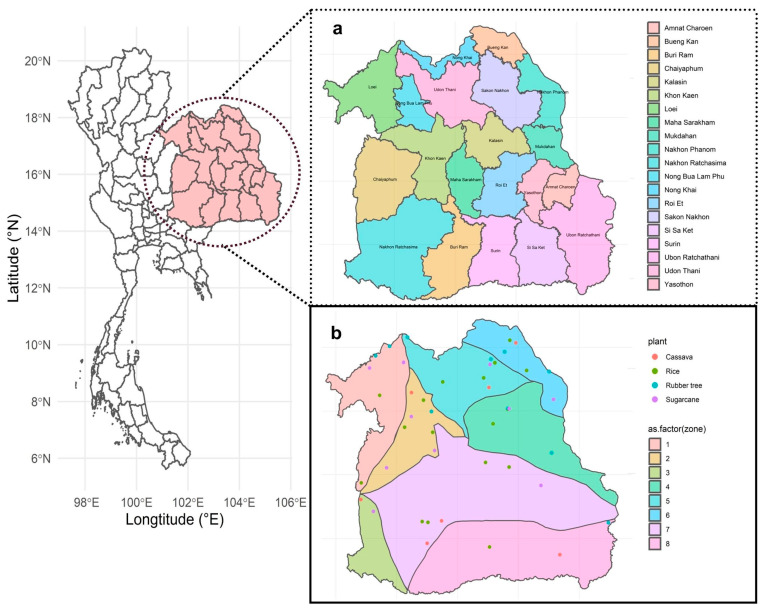
Sampling location for this experiment. (**a**) Twenty provinces in northeast Thailand. Samples were not collected from Amnat Charoen and Buriram. (**b**) Forty-six sites from four plants: Cassava (red), rice (green), rubber tree (blue), and sugarcane (purple). Eighteen province sampling sites that covered eight zones represented by different colors: zone 1 (light red), zone 2 (light gold), zone 3 (light lime green), zone 4 (light green), zone 5 (light turquoise), zone 6 (light cyan), zone 7 (light magenta), and zone 8 (light pink).

A total of 46 samples with 3 replications were collected from four plant areas of northeast Thailand from March to July 2022 (Figure 1). Samples were collected from agricultural fields in 18 different provinces, including Bueng Kan, Chaiyaphum, Kalasin, Loei, Maha Sarakham, Mukdahan, Nakhon Ratchasima, Nong Bua Lam Phu, Nong Khai, Roi Et, Sakon Nakhon, Si Sa Ket, Surin, Ubon Ratchathani, Udon Thani, and Yasothon. No samples were obtained from Amnat Charoen and Buriram provinces. The samples were collected from a depth of 5–10 cm to investigate fungi in the topsoil layer. The samples were taken near plants and were classified as bulk soil. Each point of the sample was at least 20 m apart from another point within eight zones separated by a geological feature.

The sampling area was meticulously categorized into eight distinct zones, delineated by the geological features of the plant fields (Supplementary Figure S1). These zones encompassed a range of locales, including zone 1 (Loei Petchabun), zone 2 (Phu Wiang), zone 3 (Khorat plateau), zone 4 (Phu Phan), zone 5 (Udon Sakon Nakon), zone 6 (Bueng Kan), zone 7 (Khorat Ubon), and zone 8 (Buriram). A comprehensive array of samples was gathered from various agricultural fields across eight zones, comprising 18 soil collections from rice fields, 7 from cassava fields, 11 from sugarcane fields, and 10 from rubber tree plantations (Supplementary Table S1).

### 2.2. Fungal Metabarcoding Sequencing

DNA extraction was performed using the QIAGEN DNeasy^®^ PowerSoil^®^ Pro Kit (QIAGEN, Hilden, Germany) following the manufacturer’s protocol, with slight modification by adding 60 µL of C6 (10 mM Tris elution buffer), and centrifuged at 15,000× *g* for 2 min at the last step. The first PCR amplification was performed using ITS1 as a forward primer 5′-3′ TCCGTAGGTGAACCTGCGG and ITS2 as a reverse primer 5′-3′ GCTGCGTTCTTCATCGATGC [31]. A master mix prepared for a 1X PCR contained Quanta Hifi Taq DNA polymerase 12.5 µL, ITS1 primer (10 µM) 0.5 µL, ITS2 primer (10 µM) 0.5 µL, and a total volume of 13.5 µL. Then, the extracted DNA was combined with water (PCR grade). The volume of DNA template used in the PCR reactions ranged from a minimum of 1 µL to a maximum of 11.5 µL, depending on the DNA concentration of each sample. We calculated the total DNA concentration of each sample to be approximately 100 µg/mL. A total reaction volume of 25 µ was well mixed and run on a Thermocycler. The Thermocycler conditions included starting at 98 °C for 2 min, followed by 28 cycles of denaturing at 98 °C for 20 s, annealing at 60 °C for 30 s, extension at 72 °C for 30 s, and a final extension at 72 °C for 1 min. Then, gel electrophoresis was performed by using agarose gel with a concentration of 1.2%, and the DNA fragments were visualized by using a manual gel documentation system InGenius3. After that, PCR clean-up was performed using SparQ beads (Quantabio, Beverly, MA, USA). PCR indexing was performed using Quanta Hifi Taq DNA polymerase (Quantabio, Beverly, MA, USA) 25 µL, PCR water 15 µL, Index-N 2.5 µL, Index-S 2.5 µL, and DNA template 5 µL of each sample. For the negative control, 11.5 µL of PCR-grade water was used in place of the DNA template to confirm the absence of contamination or non-specific amplification during the PCR process. For the positive control, 11.5 µL of fungal DNA from the laboratory was used to verify successful amplification. The total reaction volume was 50 µL. After mixing, PCR was performed using the following thermocycler conditions: starting at 98 °C for 2 min, followed by 10 cycles of denaturing at 98 °C for 20 s, annealing at 60 °C for 30 s, extension at 72 °C for 30 s, and a final extension at 72 °C for 1 min. Then, gel electrophoresis and PCR clean-up were performed, and DNA concentration was quantified with a fluorometer by using Qubit™ 1X dsDNA HS Assay Kits Invitrogen^®^ (Thermo Fisher Scientific Inc., Waltham, MA, USA) following the manufacturer’s protocol. DNA sequencing was performed at the Omics Sciences and Bioinformatics Center, Chulalongkorn University, using Illumina MiSeq V3 (Illumina, San Diego, CA, USA), which generated paired-end reads in 2 × 250 bp format.

### 2.3. Bioinformatics and Data Analysis

Fungal sequencing data were pre-processed using AMPtk v1.5.4 (the Amplicon Toolkit) [32]. AMPtk was used to demultiplex samples with fastq file as an input file. Output after this step was composed of an ASV table, BIOM file containing taxonomy, and metadata. The pipeline included the following steps: (1) AMPtk illumina was used to demultiplex illumina pair-end data by merging PE reads with VSEARCH [33], filtering phiX with USEARCH9 [34], rescue forward reads if pair-end reads did not merge, primer mismatch as default, and length to trim read at 230; (2) AMPtk unoise3 was the denoising algorithm used with USEARCH10 [34]; (3) AMPtk filter was used to remove index-bleed from datasets, meaning it removed read counts from the ASV table that were below the index-bleed threshold, and index-bleed was used at default 0.005; (4) AMPtk taxonomy was used to assign taxonomy using a hybrid taxonomy algorithm to ASVs and an ASV table with ITS database based on UNITE v9.0 [35]; and (5) AMPtk FUNGuild [36] was used to assign fungi ecological function information to ASVs. After pre-processing, taxonomic composition, alpha diversity, and beta diversity were analyzed to test each hypothesis by using Phyloseq packages [37] in R version 4.2.3 [38] and Rstudio version 2023.03.0 + 386 [39]. The rarefaction curve confirmed sufficient sampling depth, but the data were not rarefied, following McMurdie and Holmes’s (2014) study [37], which advised against it due to data loss. An ASV table, a taxonomy file from pre-processing with AMPtk, and a metadata file, which stores specific information of datasets, e.g., GPS coordinates (latitude and longitude), plant species, and the zone of a geological feature, were used as input files. The WHO FPPL list [1] was used as a reference to match specific taxonomy in our dataset.

## 3. Results

### 3.1. Plant Fields and Geological Features Significantly Affect Overall Fungal Richness and Communities

**Figure 2:**
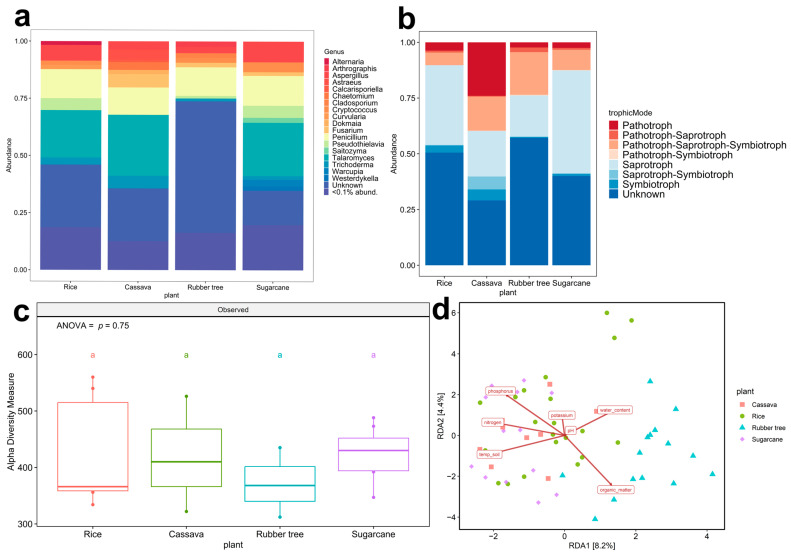
The fungal abundance from four plant fields. Zone 2 (Phu Wiang). (**a**) Fungal trophic composition (<0.1% abund. = <0.1% relative abundance) from 801 ASVs; there were 18 ASVs (genus) with a relative abundance exceeding 1 percent. The minor but diverse ASVs (<1%) represented 783 ASVs. (**b**) Fungal taxonomic composition based on the genus level; different colors indicate eight trophic modes. The unknown means unavailable ASVs ecological function in the database. (**c**) Alpha diversity observed with different colors indicated four plant fields: rice (red), cassava (green), rubber tree (blue), and sugarcane (purple). Tukey HSD significant differences (*p* < 0.05) are indicated by different letters. (**d**) Beta diversity analysis by using RDA of zone 2, zone 5 (Udon Sakon Nakon), and zone 6 (Bueng Kan).

Out of the forty-six samples collected across the eight geological zones, only zones 2, 5, and 6 were found to house all the designated plant fields. Zone 2 was characterized by a plain field geological feature with red-bed rock, while zone 5 was distinguished by a plain area with rock salt, and zone 6 featured rock salt and sandstone soil (Supplementary Table S1). The fungal taxonomic composition at the genus level across all plant fields in zone 2 (Figure 2a) provided a broad overview prior to species-level analysis and revealed the predominant presence of *Talaromyces* in all fields except for rubber tree fields. Additionally, each plant field exhibited unique genus compositions—*Alternaria* prevalent in rice fields, *Arthrographis* in rubber tree fields, *Astraeus* in cassava fields, and *Warcupia* in sugarcane fields. In zones 5 (Supplementary Figure S2a) and 6 (Supplementary Figure S2b), a greater number of genera (>1% abundance) was observed. Overall, the analysis indicates that both plant type and geological features influence fungal taxonomic composition. Fungal ecological function predictions were performed using FUNGuild to assign fungal Amplicon Sequencing Variants (ASVs) to specific trophic modes. Pathotrophs were observed across all zones in all plant fields. Cassava fields in zone 2 showed the highest abundance of pathotrophs (24.00%) (Figure 2b). In contrast, pathotroph abundance in cassava fields was lower in zone 5 (4.08%) (Supplementary Figure S2c) and zone 6 (3.48%) (Supplementary Figure S2d). In other agricultural fields, the trends of pathotroph abundance were relatively similar across all zones including sugarcane (2.50%), rubber tree (2.32%), and rice (3.73%). Saprothoph abundance was the highest in rice fields, followed by sugarcane in zones 5 and 6. In zone 2, the abundance of saprotrophs in rice fields was lower than that of sugarcane fields. The highest abundance of symbiotrophs was observed in the cassava fields of zone 2 and zone 6. However, symbiotrophs showed the highest abundance in sugarcane fields in zone 5. In summary, both plant fields and geological features impact the abundance of fungal trophic modes.

Fungal diversity was evaluated by using alpha and beta diversity analysis. The post hoc Tukey HSD test was employed for alpha diversity analysis, revealing significant differences (*p* < 0.05), as denoted by different letters. In zone 2, fungal richness showed no significant difference among plant fields (ANOVA, F (3,16) = 0.404, *p* = 0.752) (Figure 2c). However, significant differences in fungal richness by plant fields were observed in zone 5 (Supplementary Figure S3a) (ANOVA, F (3,17) = 6.045, *p* = 0.0054) and zone 6 (Supplementary Figure S3b) (ANOVA, F (3,13) = 12.96, *p* = 0.00033), in which fungal richness was lowest in rice fields and highest in sugarcane fields. The results of alpha diversity analysis indicated that the richness of fungi was significantly influenced by plant fields. Moreover, the geological features (Supplementary Figure S4) showed no effect on the fungal richness in rice, cassava, and rubber tree fields, yet it influenced sugarcane fields (*p* = 0.000017). Distinct patterns in soil properties across different plant fields were revealed by the redundancy analysis (RDA) plot (Figure 2d), with notable variations being observed in zones 2, 5, and 6, particularly separating rubber trees from other plant fields. In these zones, soil organic matter and water content emerged as influential factors, indicating their significant impact on the fungal community dynamics at the rubber tree field. Comprehensively, distinct responses to environmental factors such as temperature and soil nutrients, including nitrogen, phosphorus, and potassium that could influence fungal communities, are shown to structure fungal communities in the other three plant fields. Interestingly, the lack of significant differences in pH levels across the studied plant fields suggests that soil acidity remains relatively uniform throughout the area.

### 3.2. The Richness of WHO FPPL Pathogens Varied Among Different Plant Fields and Geological Zones

**Figure 3:**
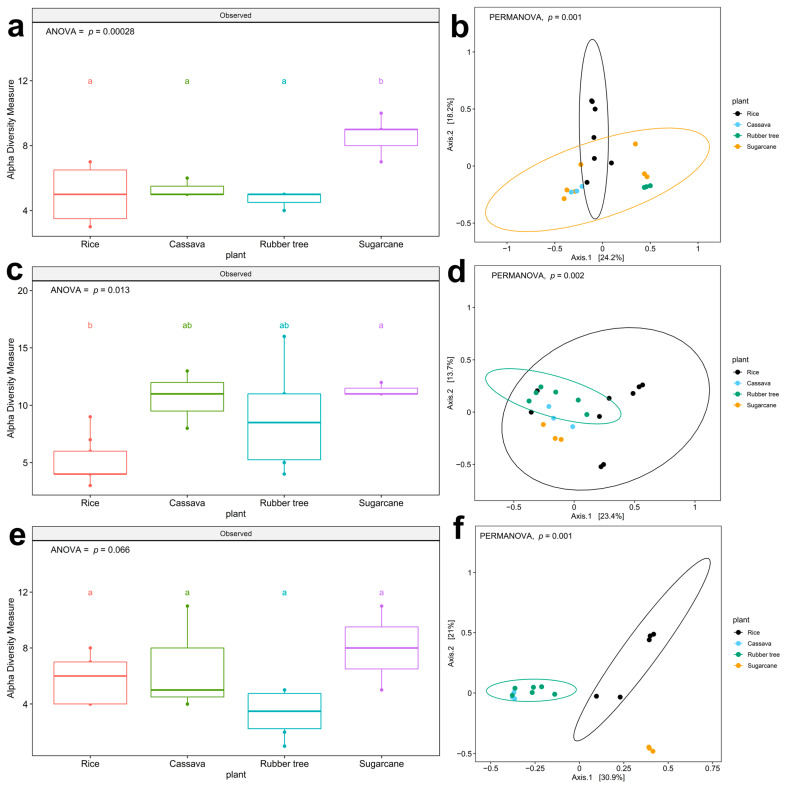
Boxplots show alpha diversity (richness) and beta diversity (PCoA) of WHO FPPL pathogens in the genus level as ASV. Richness in different plant fields: (**a**) zone 2 (**c**) zone 5, and (**e**) zone 6. Different colors indicate four plant fields: rice (red), cassava (green), rubber tree (blue), and sugarcane (purple). Boxplots show the 25th and 75th percentile, while the median is shown as lines inside the boxes, and Tukey HSD significant differences (*p* < 0.05) are indicated by different letters. Principal Coordinate Analysis in (**b**) zone 2, (**d**) zone 5, and (**f**) zone 6. Different colors indicate four plant fields, including black for rice, blue for rubber tree, green for sugarcane and yellow for cassava. Significant differences among zone 2, zone 5, and zone 6 (PERMANOVA; *p* < 0.05) were shown on PCoA.

A comprehensive analysis was conducted further for pathogens on the WHO FPPL, particularly on their ecological role as pathogens in four plant fields; alpha diversity analysis showed two patterns that were significantly different in four plant fields in zone 2 (ANOVA, F (3,16) = 11.57, *p* = 0.00028, Figure 3a) and zone 5 (ANOVA, F (3,17) = 4.848, *p* = 0.0129, Figure 3c). The richness of WHO FPPL pathogens was highest in sugarcane fields and lowest in rice fields. We observed a higher association of WHO FPPL with sugarcane fields compared to other plant fields in zone 2 (Figure 3a), where distinct clusters in Principal Coordinate Analysis (PCoA) were evident between WHO FPPL pathogens in the rice field group and those in the sugarcane field group (Figure 3b, (PERMANOVA, *p* = 0.001)). The statistical comparison revealed that WHO FPPL pathogen richness in sugarcane fields was higher and significantly different from that in rice fields. However, WHO FPPL pathogen richness in rubber tree fields and cassava fields was not significantly different. Furthermore, the WHO FPPL pathogens in zone 5 (Figure 3c,d, (PERMANOVA, *p* = 0.001)) and zone 6 (Figure 3e, (ANOVA, F (3,13) = 3.062, *p* = 0.0659) and Figure 3f, (PERMANOVA, *p* = 0.001)) showed similar clustering patterns, showing rice field clustering and rubber tree clustering, respectively. Although zone 5, characterized by a plain area with rock salt, exhibited high richness abundance in cassava and rubber tree fields, its WHO FPPL pathogens differed from those in the rice field group and rubber tree group, as depicted in Figure 3d. Conversely, the WHO FPPL pathogens in zone 6 with rock salt and sandstone soil showed high abundance in sugarcane fields, with its community group distinct from those in the rice fields and rubber trees, as shown in Figure 3f. Nonetheless, the geological features only impacted WHO FPPL pathogen richness in rubber tree fields (Supplementary Figure S4f, (*p* = 0.03)). Based on this analysis, plant fields influenced the richness of WHO FPPL pathogen abundance and dispersion, but the geological feature only affected the richness in rubber tree fields.

**Figure 4:**
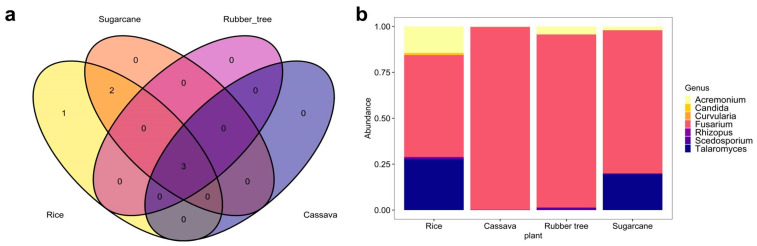
WHO FPPL pathogen distribution from four plant fields in zone 2. (**a**) Venn diagram with different colors of plant fields: rice (yellow), sugarcane (orange), rubber tree (purple), and cassava (dark blue). (**b**) Taxonomy composition based on genus level.

The Venn diagram (Figure 4a), Supplementary Figure S5a,c) visualized the number of shared and unique WHO FPPL genera in zones 2, 5, and 6. In these zones, a total of shared WHO FPPL genera was observed across four plant fields—3, 2, and 2, respectively. Thoroughly, it was supported by the taxonomic composition bar plot in zone 2, 5, and 6 (Figure 4b, Supplementary Figure S5b,d), which exhibited that *Acremonium* and *Fusarium* were found across these three zones. Other unique WHO FPPL genera associated with certain plants in specific geological zones were identified exclusively including *Candida* in rice fields of zone 2, *Curvularia* in cassava fields of zone 6, and *Candida*, *Lichtheimia*, and *Curvularia* in sugarcane fields of zone 5. These findings highlighted the impact of geographical features and plant types on WHO FPPL distribution, with similarities observed in shared WHO FPPL genera across zones and differences noted in unique WHO FPPL genera.

### 3.3. The Distribution and Documentation of WHO FPPL Pathogens Across Northeast Thailand

**Figure 5:**
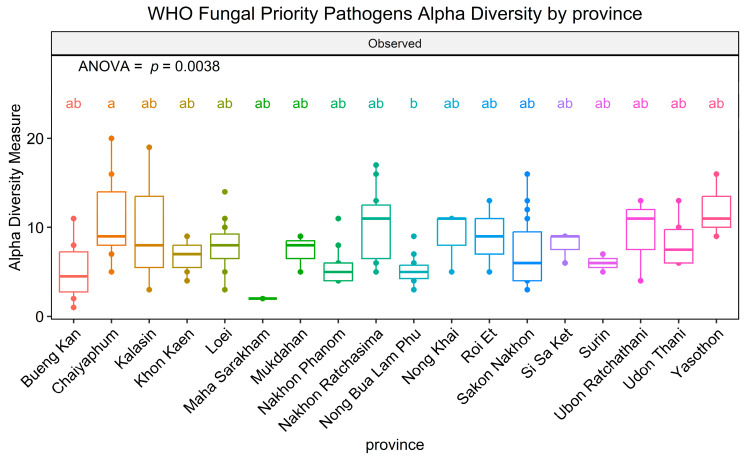
Alpha diversity of WHO FPPL pathogens in 18 provinces of northeast Thailand. Boxplots show the 25th and 75th percentile, while the median is shown as lines inside the boxes. Tukey HSD significant differences (*p* < 0.05) are indicated by different letters.

After gaining insight into the WHO FPPL pattern observed in the plant fields, the distribution of WHO FPPL pathogens in northeast Thailand was further investigated. WHO FPPL pathogen richness was detected in sugarcane and cassava fields more than in other fields. The analysis of WHO FPPL pathogen richness, based on the number of observed ASVs, indicated significant differences by province (ANOVA, F(1,17) = 2.388, *p* = 0.0038) (Figure 5). The results revealed that the richness of pathogens on the WHO FPPL was highest in Chaiyaphum (zone 2) and lowest in Nong Bua Lam Phu (zone 2). Furthermore, the richness of pathogens on the WHO FPPL was found to be highest in sugarcane fields and lowest in rice fields. Additionally, a presence/absence investigation of major WHO FPPL pathogens is presented in Table 1.

**Figure 6:**
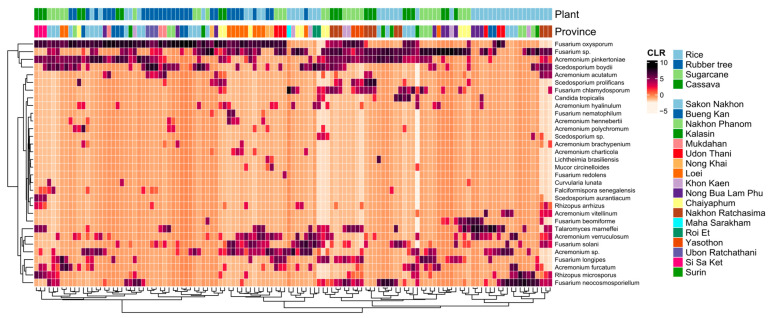
Heatmap based on the centered log-ratio (CLR) transformed values of the 32 selected fungal species of the WHO FPPL distributed in eighteen provinces in four plant fields. Plant fields present rice (light blue), rubber tree (dark blue), sugarcane (light green), and cassava (dark green).

WHO FPPL pathogens in the high- and medium-priority groups were detected, but the critical group was not found in northeast Thailand. A total of 32 fungal species on the WHO FPPL were identified in northeast Thailand soil samples including *C. tropicalis*, *Curvularia lunata*, *Falciformispora senegalensis*, *Acremonium* spp., *Fusarium* spp., *Lichtheimia* spp., *Mucor* spp., and *Rhizopus* spp. from the high-priority group and *Scedosporium* spp. and *Talaromyces marneffei* from the medium-priority group (Figure 6).

**Figure 7:**
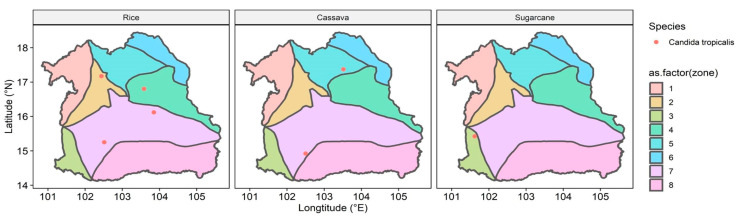
Location of *Candida tropicalis* detected in all zones of the northeastern region of Thailand in rice, cassava, and sugarcane fields. Zonation colors: zone 1 (light red), zone 2 (light gold), zone 3 (light lime green), zone 4 (light green), zone 5 (light turquoise), zone 6 (light cyan), zone 7 (light magenta), and zone 8 (light pink).

*Candida tropicalis* is one of the fungal pathogens that has been reported in the WHO FPPL, and our result showed that *C. tropicalis* was mostly detected in rice fields compared to cassava and sugarcane fields (Figure 7). Furthermore, in rice fields from zone 4, five WHO FPPL pathogens were detected alongside *C. tropicalis*, namely *Acremonium* spp., *Fusarium* spp., *Rhizopus* spp., *Scedosporium* spp., and *Mucor circinelloides* (Supplementary Figure S6). *C. tropicalis* was also identified along with *Acremonium* spp., *Fusarium* spp., *Rhizopus* spp., and *Scedosporium* spp. in cassava field samples from zone 5 and 7. Similarly, in the sugarcane fields of zone 3, *C. tropicalis* co-occurred with *Acremonium* spp., *Fusarium* spp., and *Scedosporium* spp. Additionally, our mapping also documented other WHO FPPL pathogens, including *T. marneffei*, *C. lunata*, *F. senegalensis*, and *Lichtheimia* (Supplementary Figure S6).

## 4. Discussion

Our study corroborates the notion that fungal diversity is influenced by the differences in plant fields and geological features. To the best of our knowledge, this study marks the initial examination in Thailand exploring the potential reservoirs of WHO pathogenic fungi across the entire northeastern region. The findings reveal variations in fungal communities across diverse plant fields and geological attributes. Additionally, the presence of fungal pathogens, particularly those listed in the WHO FPPL, in agricultural plant fields is highlighted. Furthermore, we identify specific distributions of WHO FPPL pathogens that warrant further investigation. Our discussion below delves into the implications of fungal communities revealed in our study, providing valuable insights for future research endeavors.

The distribution of the WHO FPPL was investigated to potentially identify the WHO FPPL reservoir and risk of exposure, particularly among farmers working in the four plant fields of northeast Thailand. While previous research at Srinagarind Hospital in Khon Kaen has primarily focused on invasive fungal infections in tertiary care settings [2], our findings suggest the presence of potential fungal pathogen reservoirs preceding these infections. This investigation provides new insight into the relationship between the WHO FPPL and agricultural plant field areas (Figure 3). The abundance of WHO FPPL pathogens in sugarcane fields was notably highest in zone 2, characterized by plain field sandstone (Figure 3a), zone 5 with a plain area of rock salt (Figure 3c), and zone 6 featuring rock salt–sandstone formations (Figure 3e). This suggests a robust association between potential WHO FPPL pathogens and sugarcane fields within regions characterized by rock salt–sandstone features; however, further investigation on actual pathogenicity is still needed. Notably, sugarcane cultivation has been linked to Fusarium’s habitat, one of the pathogens on the WHO FPPL that infected four sugarcane farmers in North India, leading to mycotic keratitis [40], and this corroborates the association that we found (Figure 4b, Supplementary Figure S5b,d). Different types of geological feature substrates are associated with specific fungal substrates, as evidenced by the presence of various genera related to the WHO FPPL in sandstone, which are *Aspergillus, Candida, Curvularia, Fusarium*, and *Rhizopus* [41], detected in zones 2 and 6. There was a lack of fungal species data associated with rock salt (halite); however, Ascomycota and Basidiomycota were found from this substrate with the dominant WHO FPPL genera being *Aspergillus*, *Sordaria*, and *Rhodotorula* [42]. Additionally, *Fusarium* spp. was observed to be widespread across zones 2, 5, and 6, encompassing four different plant fields. Furthermore, our comprehensive study identified WHO FPPL species with potential pathogenicity [18], such as *Fusarium solani* and *Curvularia lunata*, in rice fields in Chaiyaphum (zone 2) and Sakon Nakhon (zone 5) (Figure 4b and Supplementary Figure S5b). A prior study revealed that *F. solani* and *C. lunata*, common soil fungi known to cause cutaneous and subcutaneous infections in humans (eumycetoma causative), were discovered in Ubon Ratchathani, one of the provinces in northeast Thailand, associated with flies *Musca domestica* and *Chrysomya megacephala* [18]. Although there are no recorded eumycetoma cases in Ubon Ratchathani, Chaiyaphum, Sakon Nakhon, and Bueng Kan yet, this fungus was identified from clinical samples in Srinagarind Hospital, Khon Kaen [2], and Siriraj Hospital, Bangkok [43]. In a recent report from 2023, some WHO FPPL pathogens were identified as infectious fungi for immunocompromised individuals [44]. One such example is *Talaromyces marneffei*, which was also discovered in the rice fields of zones 2, 5, and 6, particularly posing a risk of infection to farmers with immuno-compromised conditions working in rice fields [17]. *T. marneffei* originates in the soil and can potentially spread to animals like field rats and humans via inhaled spores, while also attaching themselves to parts of plants, especially during the rainy season [45]. Previously, eighty patients with occupations related to agriculture and farming in northern Thailand were found to be exposed to *T. marneffei* infection, particularly those with AIDS, due to direct contact with contaminated soil [17,46]; however, managing underlying health conditions should be the primary solution rather than attempting environmental eradications. Our result also indicated that *T. marneffei* was associated with four plant fields, with the highest number of samples detected in rice fields (9 samples) and the lowest (1 sample) in cassava fields (Supplementary Figure S6f). However, the geological features were not associated with the richness of WHO FPPL pathogens, except for those in rubber tree fields (Supplementary Figure S4f), which might have been caused by the different types of plant fields compared to the other three, which were crops. Inhalation exposure among farmers has been associated with invasive fungal infections, potentially leading to conditions such as Sporotrichosis, Blastomycosis, Aspergillosis, Fusariosis, and Scedosporium infection [17], which highlights the critical need for a one health approach to address these risks. We presented WHO FPPL community environment data that can help public health workers inform about the potential reservoir and future infection risk posed by these fungal pathogens. Our study also provides documentation for research related to this scope.

The distribution of WHO FPPL pathogens across provinces in northeast Thailand suggests a higher exposure risk for workers in Chaiyaphum’s sugarcane fields compared to other areas (Figure 5). The findings from presence/absence investigations reveal that around half of these fungi could potentially be emerging pathogens (Table 1). For instance, *Mucor circinelloides*, a known cause of fatal mucormycosis [47], was exclusively detected in Kalasin, Loei, and Nong Khai provinces. Another instance involves *Acremonium* spp., where certain species have been identified in clinical samples, such as *A. egyptiacum*, but details regarding sources, like *A. charticola*, which shares similarities with *Acremonium* sp. from clinical samples, remain unclear [48]. Thus, these provinces require vigilant monitoring and management to prevent fungal spread, potentially through specific and effective antifungal treatment for infected individuals in this area or by promoting naturally antagonistic fungi. Analyzing fungal data from soil samples across various agricultural plants uncovered a consistent trend: many WHO FPPL pathogens were present in soil samples from cassava, rice, rubber, and sugarcane fields (Table 1). However, certain WHO FPPL pathogens may be specifically associated with certain plants and soils. For example, *A. charticola* was exclusively detected in rubber trees, whereas *M. circinelloides* was only detected in both rice and rubber fields. *Mucor circinelloides* and *A. charticola* have also been identified in China [49,50], with *M. circinelloides* detected in the rhizosphere of Chinese wolfsbane (*Aconitum carmichaelii*) and *A. charticola* found in the rhizosphere of cotton trees. Analysis of soil samples from various crops reveals the widespread presence of many WHO FPPL pathogens, although some are associated with specific plant types (Figure 6). For instance, *Acremonium vitellinum* was only detected in rice fields, while *M. circinelloides* occurred in both rice and rubber fields. Meanwhile, certain fungi like *Acremonium* spp., *Fusarium* spp., *Scedosporium boydii*, and *T. marneffei* were widely detected (13 out of 18 provinces, more than 72.22%), indicating common occurrence across the region. Therefore, these findings could aid in identifying potential WHO FPPL pathogens associated with each plant field, emphasizing the necessity for targeted monitoring and management strategies in these agricultural regions.

*Candida tropicalis*, detected in six provinces (33.33%), requires further investigation, particularly in key areas like Chaiyaphum and Kalasin, based on their richness significance (Figure 5). Other research showed that *Candida tropicalis* was more commonly found in tertiary care in hospitals in Thailand compared to other WHO FPPL pathogens [2]. Our findings indicate that geological features, particularly the extensive plain area with rock salt in zone 7 of the northeastern region, might impact the distribution of pathogens like *C. tropicalis*. This yeast, known for its moderate halotolerance [51], is more likely to be found in zones with rock salt, such as zone 7. *Candida tropicalis*, identified as a high-priority fungal pathogen [1], showed varying abundance across different geological features and was more frequently detected in rice fields, followed by cassava and sugarcane fields (Figure 7). *Candida* species represent a common cause of bloodstream infection (BSI) in humans, and recently *C. tropicalis* was identified as the most common cause of candidemia associated with high mortality in Bangkok [52,53]. Our findings indicate that individuals involved in rice fields, particularly in northeast Thailand, should be mindful of *C. tropicalis*. Nonetheless, this serves as a cautionary note for rice farmers in other regions and countries as well, especially those that have soil with moderate salinity.

Another result of fungal communities among various plant fields is based on their ecological role, such as symbiotroph to plant and pathotroph. An illustration of a plant symbiotroph is plant growth-promoting fungi (PGPF), which are part of the microbial hubs discovered in the rhizosphere of normal fields and serve as mediators for various microbial functions within the rhizosphere environment [52]. These PGPF, which belong to the Ascomycota phylum [54,55], including *Aspergillus* (2.76 to 9.03%), *Chaetomium* (0.02 to 3.42%), *Cladosporium* (0.01 to 2.01%), *Fusarium* (0.20 to 5.84%), *Penicillium* (11.95 to 13.03%), *Talaromyces* (0.43 to 26.66%), and *Trichoderma* (1.23 to 5.49%), were also found in four plant fields of zone 2 (Figure 2a). *Aspergillus*, *Cladosporium*, *Fusarium*, *Penicillium*, and *Trichoderma* possess the capability to stimulate the production of phytohormones [55]; for instance, the combination of *Aspergillus fumigatus* TS1 and *Fusarium proliferatum* BRL1 has been shown to produce gibberellin and indole-3-acetic acid (IAA), influencing rice root development [56]. Nonetheless, soil fungal communities play diverse roles, including those of fungal pathogens [57], which we identified across four plant fields as pathotrophs (Figure 2b); additionally, the distribution of fungal communities in zone 2 exhibited a uniform dispersion across each plant field, as depicted in Figure 2c. Furthermore, the observations of abiotic factors are depicted in Figure 2d, aligning with findings from related research that demonstrated how the fungal diversity in cassava and rubber tree fields has shared and unique communities [58]. The investigation into the arbuscular mycorrhizal fungi (AMF) communities between rubber trees and cassava revealed nuanced differences driven by soil texture and nutrient composition—specifically, that potassium levels and silt soil are pivotal in molding fungal communities in cassava fields, whereas phosphorus and organic matter contents along with sand soil predominantly shape those in rubber tree fields [58]. Our findings indicate that nitrogen, phosphorus, and soil temperature significantly impact cassava fields, whereas organic matter is a major factor influencing rubber tree fields (Figure 2d). Moreover, from fungal communities of the biochar application to the rubber soil experiment, we examined the impact of pH and phosphorus levels on fungal communities [59], although these factors did not significantly affect our findings (Figure 2d). In our results, we observed that phosphorus affected all the fungal communities in cassava fields, which contrasts with another study’s finding that phosphorus had no impact, except on arbuscular mycorrhizal fungi communities [60]. Organic matter is pivotal for nurturing fungal community growth in sugarcane fields, while nitrogen and phosphorus also contribute to this dynamic [61], aligning closely with conditions observed in our sampling sites in sugarcane fields (Figure 2d). The fertilization farming system, in sugarcane fields with sandy soils in Nakhon Ratchasima, Buriram, and Khon Kaen, had a notable impact on the presence of AMF [61]. Soil phosphorus levels impacted the composition of AMF communities in rice fields, leading to increased colonization of roots by AMF and subsequently enhancing rice growth [62]; this factor also shaped the fungal communities observed in our rice field samples (Figure 2d). The geological feature factor only affected the richness of the fungal community in sugarcane fields at zone 2; this might have been caused by fungal adaptability based on the substrate that was associated with the four plants being different—for instance, sandstone in zone 2 compared to salt rock in zone 5 and mixed sandstone with salt rock in zone 6 (Supplementary Table S1). A total of 36 genera were found in sandstone as a substrate [41], and the dominant genera found in rock salt were *Aspergillus*, *Sordaria*, and *Rhodotorula* [42]. In addition, farmers commonly apply fertilizer to optimize crop yield to gain soil nutrients, and that is becoming a significant factor shaping fungal diversity in rice fields [63]. Moreover, the dynamic of fungal communities is impacted by human land use [64] and vegetation conditions [65]. This evidence supports the hypothesis that fungal communities differ across plant fields in connection with geological features.

While our study offers valuable insights, it is important to acknowledge its limitations within the one health framework. We identified WHO FPPL pathogens in the high- and medium-priority groups but did not detect any in the critical group. Relying solely on next-generation sequencing may have limited our ability to accurately identify some fungal species, as isolation techniques were not used. Our results are based on predictive models of fungal communities, indicating potential rather than definitive infections among farmers. Nonetheless, our study represents a significant step forward, covering a broader range of areas and providing a comprehensive map of WHO FPPL fungal pathogen locations. It also serves as essential documentation for ongoing research. This information is invaluable for future research [66] and highlights the interconnectedness of human, animal, and environmental health. Identifying fungal pathogens in agricultural fields is particularly relevant for farmers, who are exposed at the frontline of potential zoonotic and infectious diseases. Proper hand hygiene, including regular handwashing after handling soil, plants, or organic materials, is advised to prevent pathogen transfer to mucous membranes or open wounds. Additionally, the use of personal protective equipment (PPE), particularly rain boots, is recommended to minimize health risks, especially for individuals with foot wounds. By implementing these measures, exposure risks can be reduced, and safer working conditions in agricultural settings can be promoted. Furthermore, Integrated Pest Management (IPM) and organic agriculture may indirectly reduce fungal pathogen prevalence by minimizing chemical use [67]. Our findings underscore the need for vigilant surveillance and continued research efforts in Thailand. Integrating environmental, agricultural, and health data is essential for developing effective strategies to prevent and control fungal infections, ultimately protecting the health of humans, animals, and ecosystems.

## 5. Conclusions

In conclusion, our study represents a pioneering endeavor in thoroughly investigating fungal diversity and identifying potential WHO FPPL pathogens across soil agricultural areas in northeast Thailand. Our investigation demonstrated that the richness of both general fungal communities and WHO FPPL communities are influenced by different plant fields and geological features. Additionally, the distribution of WHO FPPL pathogens varies across northeast Thailand, with notably high abundance observed in sugarcane fields in Chaiyaphum and *C. tropicalis* mostly found in zones with rock salt. To our knowledge, this is the first comprehensive survey covering the entirety of the region’s agricultural soil. By mapping potential reservoirs of the WHO FPPL reservoir as of 2023, we provide essential data for public health workers, enabling better-informed decisions to manage and mitigate exposure to these fungal pathogens and connect them to the one health framework. This comprehensive documentation underscores the importance of public awareness and prompt management procedures to reduce the risk of WHO FPPL infections and diseases in agricultural communities. Our research thus serves as a crucial step toward integrating environmental surveillance with human and animal health strategies, fostering a holistic approach to preventing and controlling fungal diseases. Lastly, future studies should investigate the survival of these fungi in field conditions, examine their potential transmission routes, and apply functional analyses such as metagenomics to explore their metabolic activity. Combining clinical surveillance with different seasons and environmental conditions will provide better insights into the connection between fungal presence in agricultural fields and its impact on human health.

## Figures and Tables

**Table 1:** WHO FPPL pathogens in soil samples from different agricultural fields that were found only in particular plant–soil.

WHO FPPL	Cassava	Rice	Rubber Tree	Sugarcane
*Acremonium charticola* ^a^	-	-	Present	-
*A. hennebertii* ^a^	-	Present	Present	-
*A. vitellinum* ^a^	-	Present	-	-
*Curvularia lunata* ^a^	Present	-	-	Present
*Fusarium nematophilum* ^a^	-	Present	Present	-
*F. redolens* ^a^	-	-	-	Present
*Lichtheimia brasiliensis* ^a^	-	Present	Present	-
*Mucor circinelloides* ^a^	-	Present	Present	-
*Scedosporium* sp. ^b^	-	Present	-	Present
*S. aurantiacum* ^b^	Present	Present	-	-

^a^ High-priority group; ^b^ Medium-priority group.

## Data Availability

Raw sequence reads were submitted to the Sequence Read Archive databases associated with BioProject accession number BioProject ID: PRJNA1233617.

## Supplementary Materials

The following supporting information can be downloaded at https://www.mdpi.com/article/10.3390/life15030488/s1, Figure S1: The four plant field compositions from eight geological features: Zone 1 (Loei Petchabun), zone 2 (Phu Wiang), zone 3 (Khorat plateau), zone 4 (Phu Phan), zone 5 (Udon Sakon Nakon), zone 6 (Bueng Kan), zone 7 (Khorat Ubon), and zone 8 (Buriram). Different color of plant fields: Rice (red), cassava (blue), rubber tree (green), and sugarcane (purple); Figure S2: Taxonomic composition bar plot zone 5 (**a**) 31 genera and zone 6 (**b**) 32 genera of 801 ASVs, with <0.1% abund. equal to <0.1% relative abundance. Fungal trophic bar plot zone 5 (**c**) and zone 6 (**d**). The unknown means unavailable ASVs ecological function in the database; Figure S3: Alpha diversity of fungal community in zone 5 (**a**) and zone 6 (**b**) with boxplots show 25th and 75th percentile while median was shown as lines inside boxes and Tukey HSD significant differences (*p* < 0.05) are indicated by different letters. Different colors indicated four plant fields: Rice (red), cassava (green), rubber tree (blue), and sugarcane (purple); Figure S4: Alpha diversity of fungal community from four plant fields based on the three zones, zone 2 (Phu Wiang), zone 5 (Udon Sakhon Nakon) and zone 6 (Bueng Kan). Boxplots show 25th and 75th percentile while median was shown as lines inside boxes and Tukey HSD significant differences (*p* < 0.05) are indicated by different letters. The plant fields; Rice (**a**) for general fungal community and (**b**) for WHO FPPL; Cassava (**c**) for general fungal community and (**d**) for WHO FPPL; Rubber tree (**e**) for general fungal community and (**f**) for WHO FPPL; Sugarcane (**g**) for general fungal community and (**h**) for WHO FPPL; Figure S5: The WHO FPPL distribution from four plant fields. Venn diagram (**a**) zone 5 and (**c**) zone 6 with different color of plant fields: Rice (yellow), sugarcane (orange), rubber tree (magenta), and cassava (dark blue). Taxonomy composition based on genus level in four plant fields in (**b**) zone 5 and (**d**) zone 6; Figure S6: Map distribution of WHO FPPL in eight zones of northeast Thailand: Zone 1 (light red), zone 2 (light gold), zone 3 (light lime green), zone 4 (light green), zone 5 (light turquoise), zone 6 (light cyan), zone 7 (light magenta), and zone 8 (light pink). (**a**) *Acremonium* spp., (**b**) *Falciformispora senegalensis*, (**c**) *Fusarium* spp., (**d**) *Rhizopus* spp., (**e**) *Scedosporium* spp., (**f**) *Talaromyces marneffei*, (**g**) *Curvularia lunata,* (**h**) *Lichtheimia*, and (**i**) *Mucor circinelloides*; Table S1: The number of sampling sites in the zone that includes province area based on geological features and four plant fields.
