# Supplementary material for: Exploring Fungal Abundance and WHO Fungal Priority Pathogens in Agricultural Fields: A One Health Perspective in Northeast Thailand
Source: Life (Basel). 2025 Mar 18;15(3):488. doi: 10.3390/life15030488 (PMC11944233; doi:10.3390/life15030488)
Supplement: Supplementary file 1 [file life-15-00488-s001.zip › Table S1.pdf]

Supplementary Table S1. The number of sampling sites in the zone that includes province area based on geological features and four plant fields.

Zone	Description	Province	Geological Features	Rice	Cassava	Sugarcane	Rubber tree
Zone 1	Loei-Petchabun Fold belt	Loei, Khon Kaen, Chaiyaphum, and Udon Thani	Mountain range, mostly sedimentary rock (granite)	6	0	6	6
Zone 2	Phu Wiang area	Nong Bua Lam Phu, Khon Kaen, and Chaiyaphum	Plain area with flat-topped mountains, red-bed rock (sandstone)	7	3	7	3
Zone 3	Khorat Plateau's Edges	Chaiyaphum and Nakhon Ratchasima	Sandstone mountains	0	3	2	0
Zone 4	Phu Phan Mountain range	Sakon Nakhon, Kalasin, and Mukdahan	Mountain range, Khorat Group sedimentary rock (sandstone, conglomerate, siltstone, shale, claystone, and rock salt)	6	0	3	5
Zone 5	Udon Sakon Nakon area	Sakon Nakhon, Udon Thani, and Nong Khai	Plain area, rock salt present	9	3	3	6
Zone 6	Bueng Kan area	Bueng Kan and Nakhon Phanom	Rock salt and sandstone present	5	3	3	6
Zone 7	Khorat-Ubon area	Yasothon, Roi Et, Maha Sarakham, and Nakhon Ratchasima	Geologically like Zone 5 but larger	8	6	3	0
Zone 8	Buriram area	Surin, Si Sa Ket, and Ubon Ratchathani	Geologically like Zone 6 but lacks rock salt and sandstone	3	3	0	3
