# Supplementary figures and images for: Exploring Fungal Abundance and WHO Fungal Priority Pathogens in Agricultural Fields: A One Health Perspective in Northeast Thailand
Source: Life (Basel). 2025 Mar 18;15(3):488. doi: 10.3390/life15030488 (PMC11944233; doi:10.3390/life15030488)

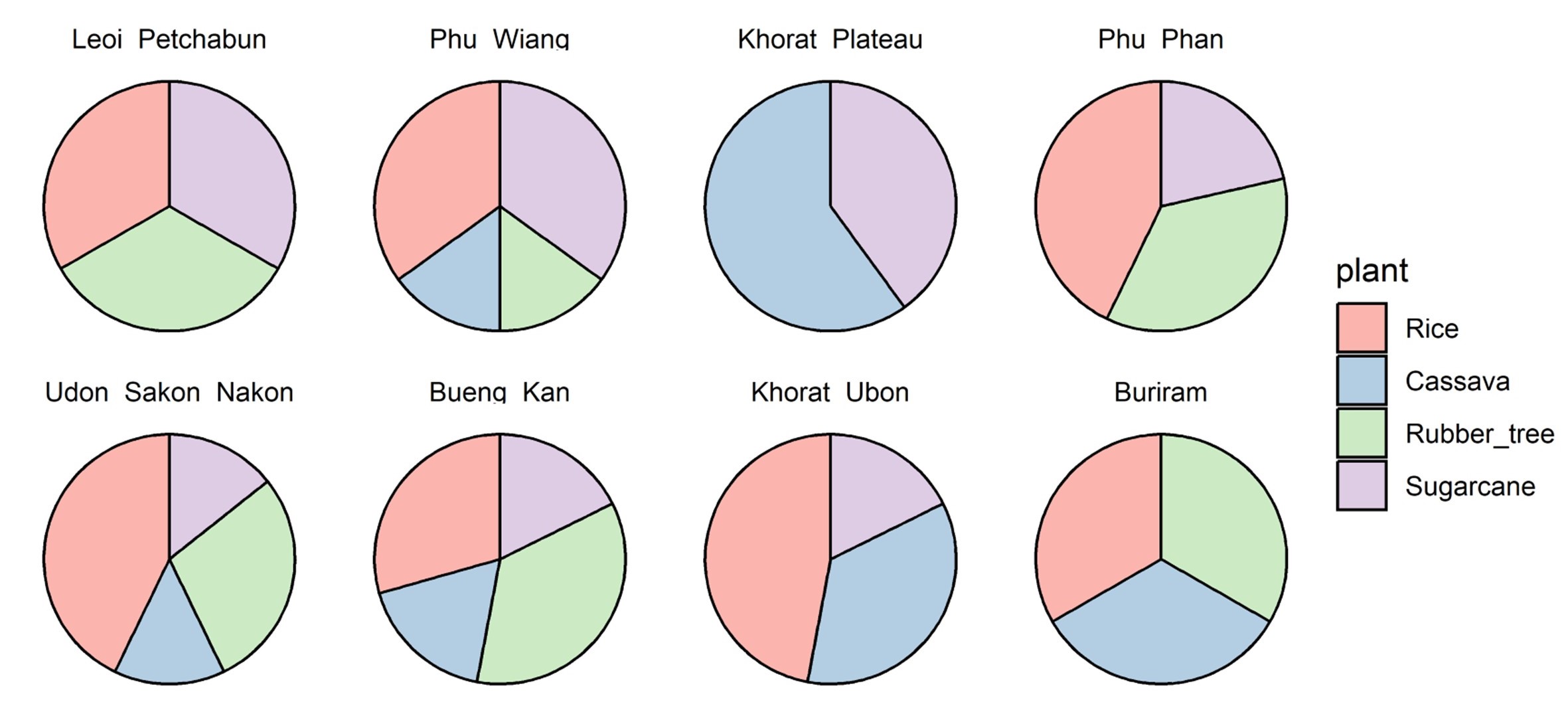

Supplement: Supplementary file 1 [file life-15-00488-s001.zip › supplementary Figure S1.jpg]

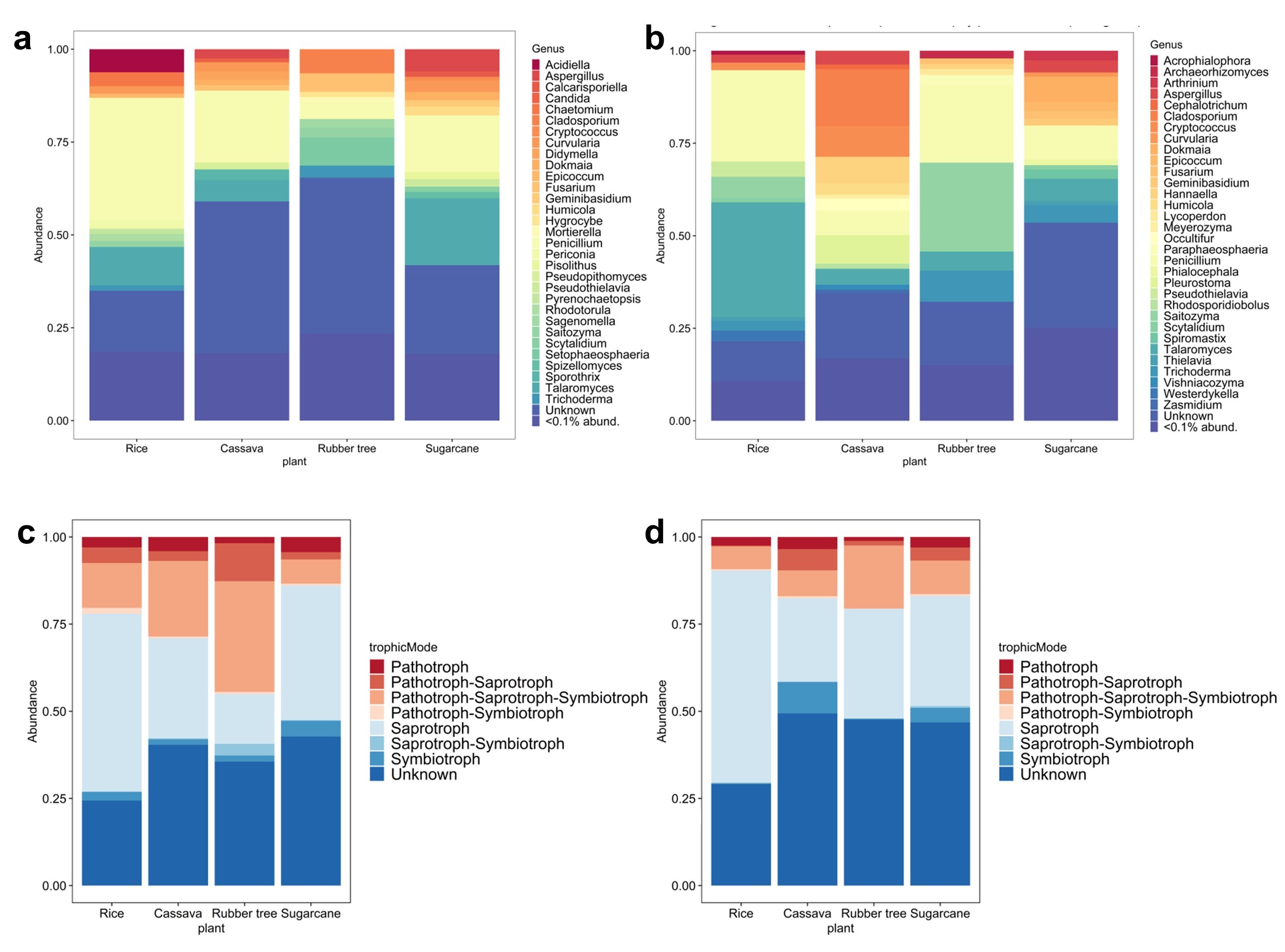

Supplement: Supplementary file 1 [file life-15-00488-s001.zip › supplementary Figure S2.jpg]

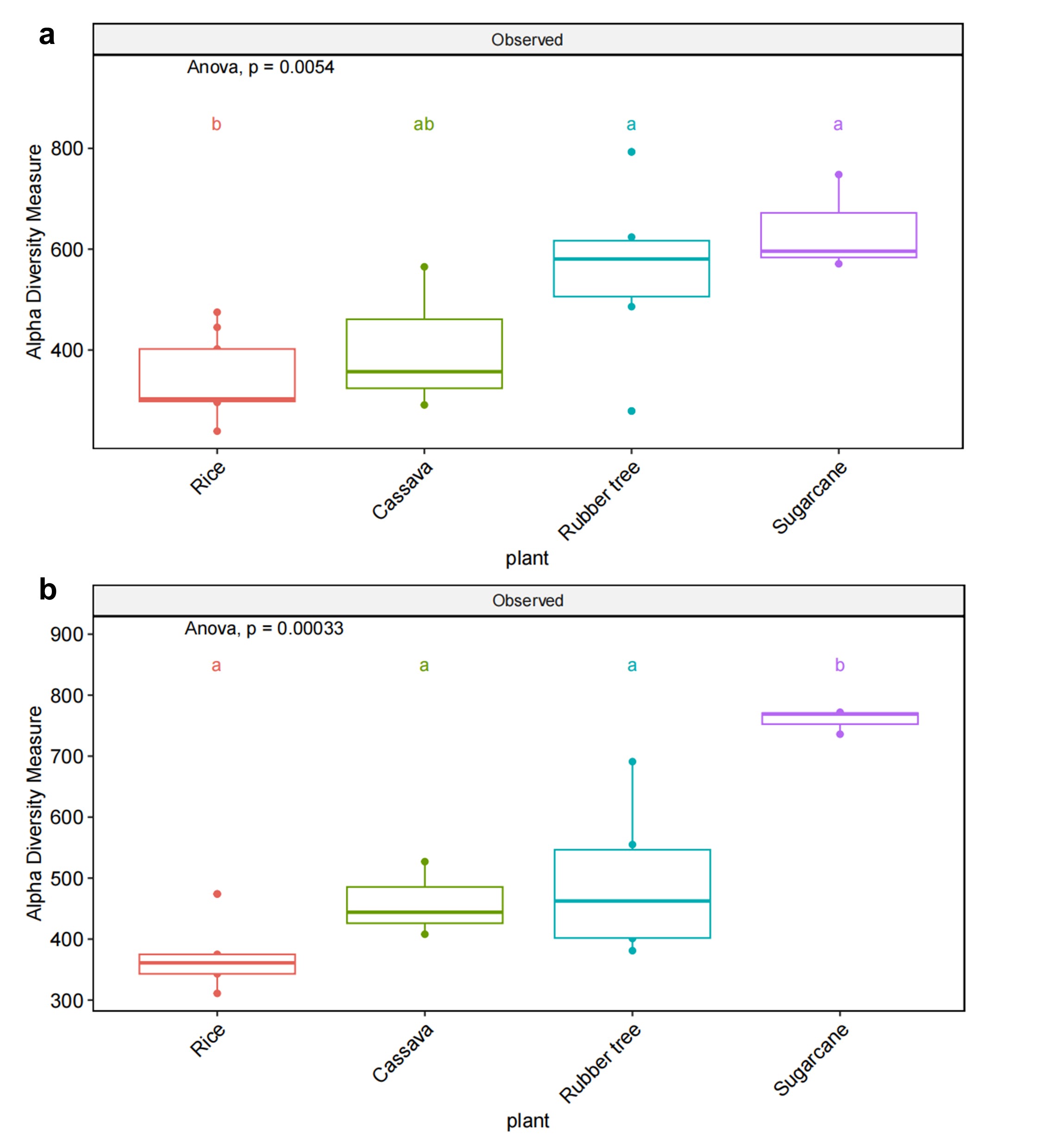

Supplement: Supplementary file 1 [file life-15-00488-s001.zip › supplementary Figure S3.jpg]

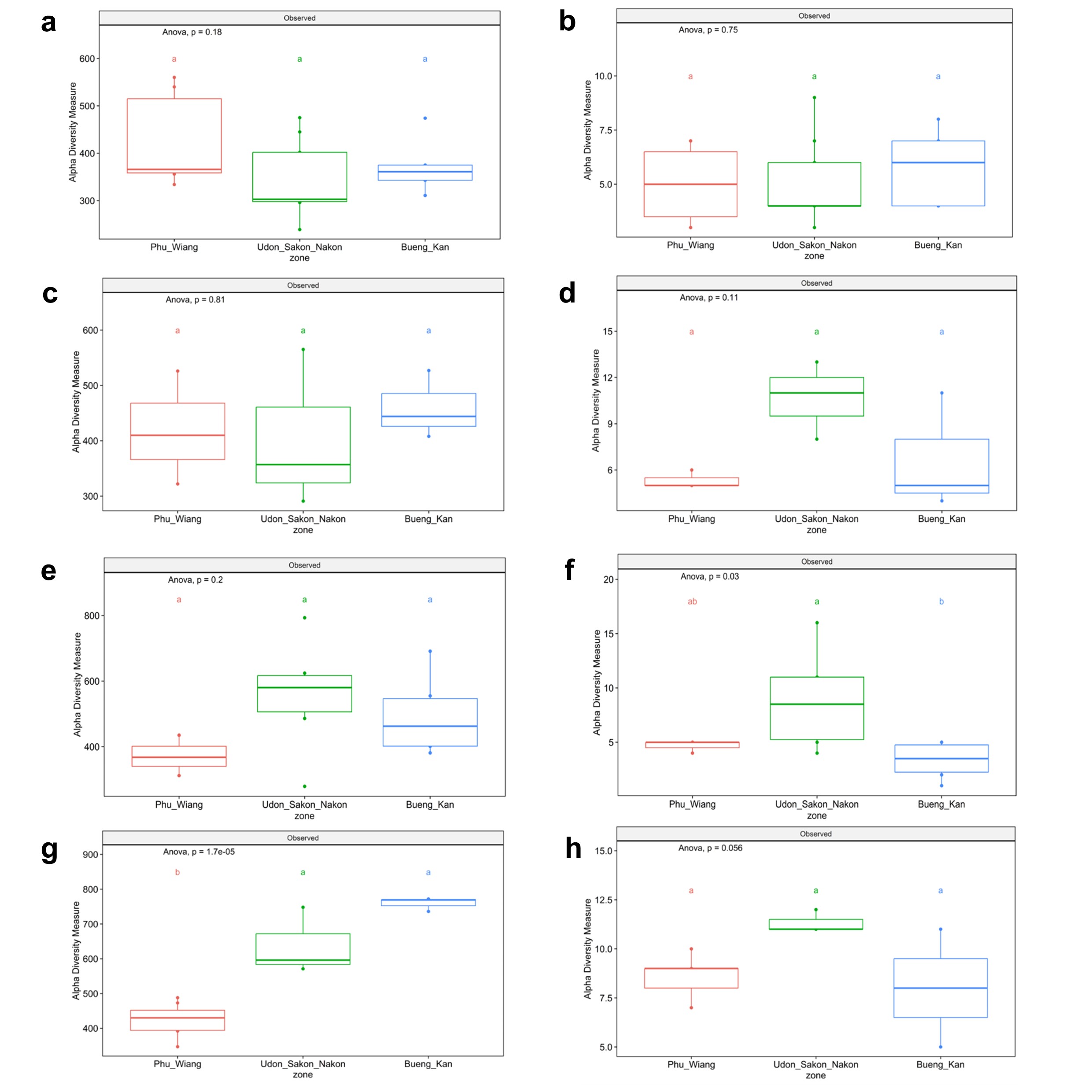

Supplement: Supplementary file 1 [file life-15-00488-s001.zip › supplementary Figure S4.jpg]

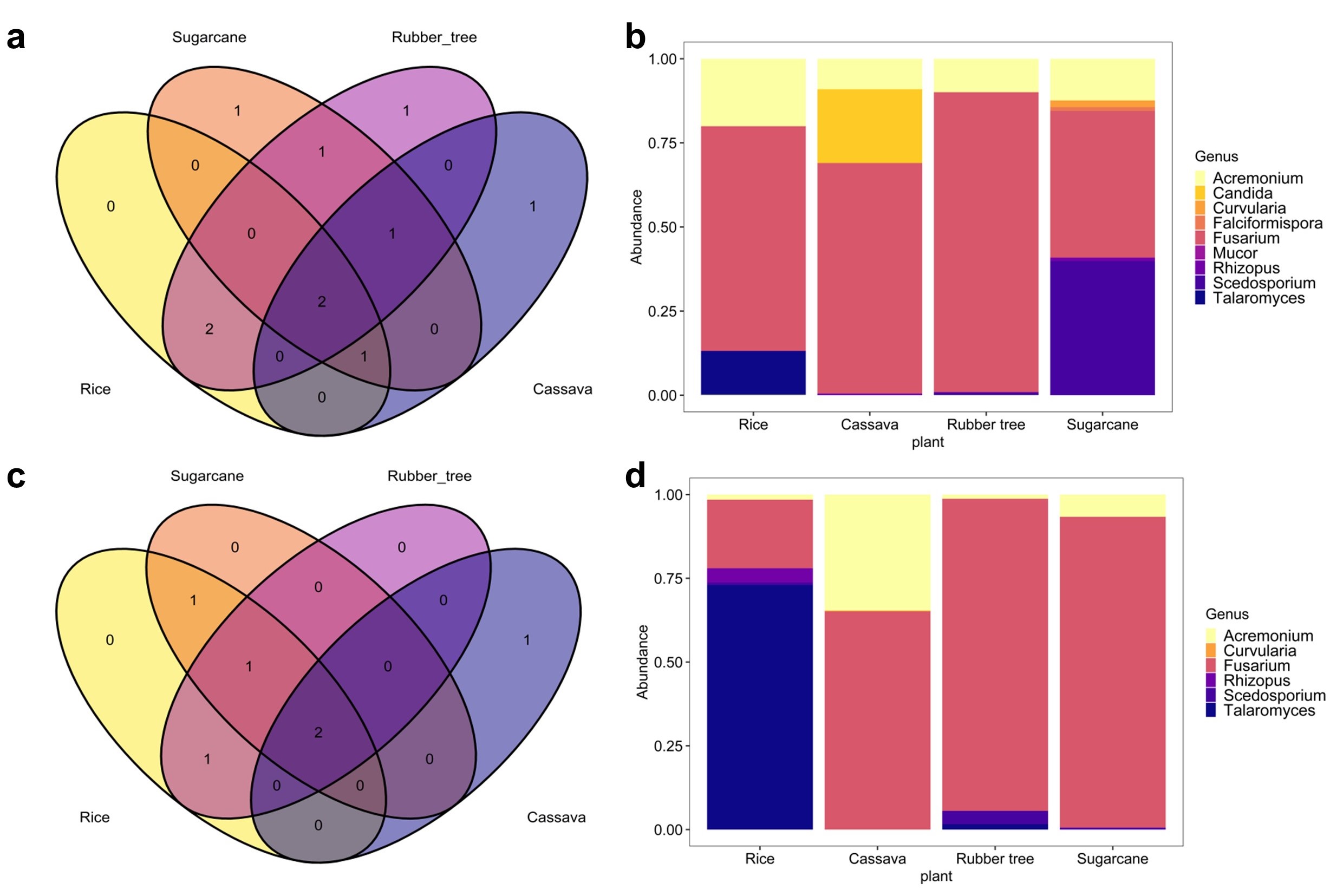

Supplement: Supplementary file 1 [file life-15-00488-s001.zip › supplementary Figure S5.jpg]

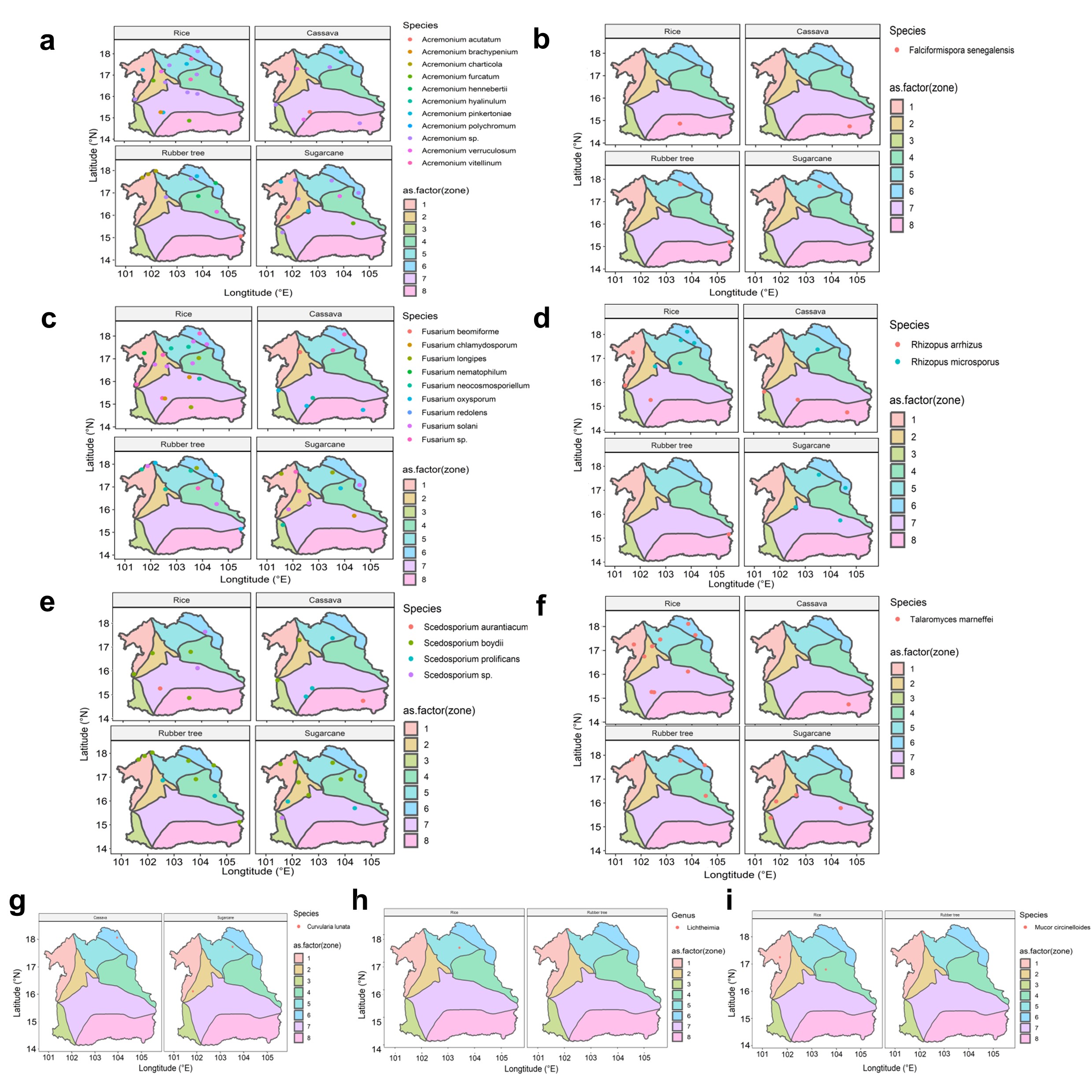

Supplement: Supplementary file 1 [file life-15-00488-s001.zip › supplementary Figure S6.jpg]
